# Physical Activity Counseling for Adults with Hypertension: A Randomized Controlled Pilot Trial

**DOI:** 10.3390/ijerph17176076

**Published:** 2020-08-21

**Authors:** Altieres E. Sousa Junior, Geovani A. D. Macêdo, Daniel Schwade, Júlio Sócrates, José W. Alves, Luiz F. Farias-Junior, Yuri A. Freire, Telma M. A. M. Lemos, Rodrigo A. V. Browne, Eduardo C. Costa

**Affiliations:** 1 Graduate Program in Physical Education, Federal University of Rio Grande do Norte, Natal 59072-970, RN, Brazil; altieresjunior21@hotmail.com (A.E.S.J.); g.macedoef@gmail.com (G.A.D.M.); wifisonalves@outlook.com (J.W.A.); yuriadg@gmail.com (Y.A.F.); 2 Department of Physical Education, Federal University of Rio Grande do Norte, Natal 59072-970, RN, Brazil; danielschwadea@gmail.com; 3 Graduate Program in Health Sciences, Federal University of Rio Grande do Norte, Natal 59012-570, RN, Brazil; juliosocratess@gmail.com (J.S.); rodrigodenatal@gmail.com (R.A.V.B.); 4 Graduate Program in Psychobiology, Federal University of Rio Grande do Norte, Natal 59064-741, RN, Brazil; lfariasjunior@gmail.com; 5 Department of Clinical and Toxicological Analysis, Federal University of Rio Grande do Norte, Natal 59012-570, RN, Brazil; telmaml@yahoo.com.br

**Keywords:** exercise, cardiovascular health, pedometry, sitting/standing, health behavior

## Abstract

The effect of physical activity counseling (PAC) in hypertensive adults is unclear. This study investigated the effect of PAC on blood pressure (BP), physical activity level, sitting time, metabolic profile, and body composition in hypertensive adults. Twenty-two hypertensive adults (48.8 ± 7.3 years) participated in this pilot trial. The 12-week PAC was based on the 5 A’s model considering the FITT principle (Frequency, Intensity, Time, and Type) of physical activity. The control group received instructions about FITT in one face-to-face meeting at baseline. Pedometer-measured physical activity, sitting time, resting and ambulatory BP, metabolic profile (cholesterol, triglycerides, fasting glucose), and body composition (fat mass, abdominal fat, fat free mass) were assessed. The PAC group showed higher steps per day (5839 ± 992 vs. 5028 ± 902; *p* = 0.044) and a trend for lower sitting time (5.6 ± 1.3 vs. 8.0 ± 4.0 h/day; *p* = 0.059) than the control group. No changes were observed in BP, metabolic profile, and body composition (*p >* 0.05). In conclusion, 12 weeks of a PAC program based on the 5 A’s model resulted in a modest increase of ~800 steps per day and a trend to decrease ~2 h/day in sitting time, but there were no associated reduction in BP and improvements in metabolic and body composition.

## 1. Introduction

Hypertension is a major risk factor for cardiovascular disease (CVD) and mortality [1,2] with a prevalence of ~40% worldwide [3]. Pharmacological and non-pharmacological treatments are recommended as therapeutic approaches for hypertension [4]. Physical exercise is a cornerstone for the non-pharmacological treatment of hypertension [4,5]. Randomized controlled trials have reported a BP-lowering effect of aerobic exercise in both resting [6,7] and ambulatory BP [8]. A network meta-analysis reported that the BP-lowering effect of aerobic exercise is similar to antihypertensive medications [9]. However, most hypertensive individuals do not meet the physical activity recommendations [10].

In order to increase the physical activity level, professional committees and organizations [11,12,13] have suggested the implementation of physical activity counseling (PAC) programs in clinical and public health settings. A previous systematic review [14] showed that PAC interventions are able to increase the physical activity level of healthy adults. Lin et al. [15] showed that PAC programs associated with dietary interventions are able to reduce 2 mmHg of systolic and 1.4 mmHg of diastolic BP in adults with CVD risk factors. In hypertensive population, PAC seems to be a promising approach to improve BP control and health-related outcomes, although more data are needed. Lee et al. [16] found that a six-month walking intervention elicited a decrease of 7 mmHg of systolic BP in hypertensive older adults, while Farinatti et al. [17] found that 8 months of a home-based PAC decreased 4 mmHg of systolic BP in middle-aged women with hypertension.

Counseling interventions based on the 5 A’s model (i.e., Assess, Advice, Agree, Assist, and Arrange), which is a patient-centered counseling approach [18,19,20], have been used to improve health and induce weight loss in overweight/obese individuals [21], treat alcohol abuse, smoking addiction, dyslipidemia [22,23], and to increase physical activity [24]. To the best of our knowledge, no information is available about the effect of a PAC program based on the 5 A’s model on physical activity level and BP status in hypertensive adults. Therefore, the primary aim of this study was to investigate the effect of a PAC program based on the 5 A’s model on physical activity level and BP status (resting and ambulatory) in hypertensive adults. Additionally, we investigated the effect of this intervention on sitting time, metabolic profile and body composition as secondary objectives.

## 2. Materials and Methods

### 2.1. Study Design

This randomized controlled pilot trial is reported in accordance with the Consolidated Standards of Reporting Trials (CONSORT) statement [25]. The following assessments were conducted after the initial screening at baseline: (i) pedometer-measured physical activity level; (ii) 24-h ambulatory BP monitoring; (iii) metabolic profile [total cholesterol, high density lipoproteins (HDL-cholesterol), low density lipoproteins (LDL-cholesterol), triglycerides, and fasting glucose]; (iv) body composition assessed by dual-energy X-ray absorptiometry (DXA). Participants were randomized into two groups after the baseline assessments: (i) The PAC program based on the 5 A’s model; or (ii) control group. The study was conducted from August 2016 to May 2017 at the Hospital and at the Department of Physical Education of University. The Institutional Ethics Committee approved the study (protocol no. 1.537.438/2016) and the conduct of the study conformed to the ethical principles outlined in the Declaration of Helsinki.

### 2.2. Participants

**Figure 1:**
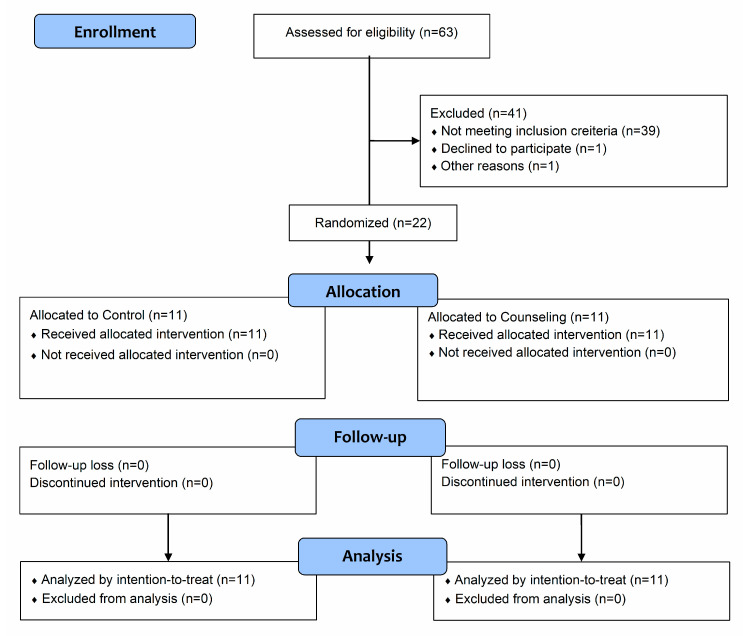
Flow diagram of the study.

Twenty-two hypertensive adults participated in this study (Figure 1). Participants were recruited from the university cardiology outpatient service of the University Hospital and via social media. Inclusion criteria were: age from 30 to 59 years; previous diagnosis of hypertension; currently taking antihypertensive medication(s); no participation in exercise training programs in the last six months; being ‘inactive’; i.e., less than 7500 steps/day; no known cardiovascular, metabolic, renal or respiratory disease; and no musculoskeletal conditions that limit their ability to exercise. Exclusion criteria were: high resting BP levels (i.e., ≥160/105 mmHg); pain or injury which limits ability to exercise during the study procedures; and changes in antihypertensive medication(s) during the study.

### 2.3. Screening Visit

Participants were screened upon their first visit using the Physical Activity Readiness Questionnaire [26] medical history, and medication use. Body mass (kg) and height (m) were measured using a calibrated digital scale attached to a stadiometer (Welmy^®^, W300, Santa Bárbara d’Oeste, SP, Brazil). Body mass index (BMI) was calculated as the ratio between weight and height squared (kg/m^2^) [27]. HR (Polar Electro^®^, Kempele, Oy, Finland) was measured following 10 min resting in a seated position [28]. Resting BP was measured in a seated position using an oscillometric device (Omron^®^ HEM-780-E, Shimogyo-ku, Kyoto, Japan) in triplicate with 1-min intervals between each measure. The average value of the last two measures was recorded [2]. Afterward, the participant’s body composition was assessed by DXA (GE Healthcare Lunar Prodigy Advance^®^, Chicago, IL, USA). At the end of the screening visit, participants were fitted with a wrist pedometer (Omron^®^, HJ-321 Tri-Axis Alvita, Cook County, IL, USA) to determine their physical activity level. The participants came back to the laboratory one week later to remove the pedometer and perform blood sampling to assess the metabolic profile. Finally, participants were fitted with an ambulatory BP device (CardioMAPA, Cardios^®^, São Paul, Brazil) in order to determine their ambulatory BP over a 24-h period.

### 2.4. Pedometer-Measured Physical Activity Level and Sitting Time

Pedometers (Omron^®^, HJ-321 Tri-Axis, Shimogyo-ku, Kyoto, Japan) were individually adjusted for the participants based on their stride length, weight and height according to the manufacturer’s instructions. A total number of steps per day less than 7500 was used as a criterion to classify the participants as ‘inactive’ [29]. The total number of steps per day was determined over a 1-week period in order to assess the pedometer-measured physical activity level. Sitting time (h/day) was assessed during week and weekend days using a self-reported questionnaire [30] as a proxy of sedentary behavior. The average value of sitting time during week and weekend days [(∑weekdays × 5) + (∑weekend days × 2) ÷ 7] was used for data analysis.

### 2.5. Ambulatory Blood Pressure Monitoring

Participants were fitted with an ambulatory BP monitoring device (CardioMAPA, Cardios^®^, São Paulo, SP, Brazil) on their non-dominant arm [31]. The device was programmed to measure BP every 15 min during the awake period and every 30 min during the sleep period. A minimum of 16 and eight BP measurements during awake and sleep periods, respectively, had to be successfully recorded to be included in the final analysis [31]. Data was recorded during a 24-h period with the awake and sleep periods defined according to each participant. The average values for each period were considered for data analysis. 

### 2.6. Metabolic Profile

Blood samples were collected by venipuncture after a 12-h overnight fasting period. The metabolic profile included total cholesterol, HDL-cholesterol, LDL-cholesterol, triglycerides, and fasting glucose. All biochemical assays were conducted by an automatic biochemical analyzer using commercial kits (Labmax Plenno, Labtest^®^, Belo Horizonte, Brazil), according to the manufacturer’s instructions. LDL-cholesterol levels were determined by the Friedewald formula: (total cholesterol – [HDL-cholesterol + triglycerides/5]) [32].

### 2.7. Physical Activity Counseling Program

The PAC program was based on the 5 A’s model, which is a patient-centered counseling approach [18,19,20]. The participants from this group participated in six individual face-to-face PAC meetings biweekly over the 12-week period. Participants were counseled about the FITT (Frequency, Intensity, Time, and Type) ‘dose’ recommended for health promotion according to physical activity guidelines [33] and progressive goals were established every visit with agreement between the participant and researcher (Box 1).

Box 1 **Physical activity counseling goals over the 12 weeks of intervention in adults with hypertension according to the FITT principle (Frequency, Intensity, Time, Type).**

**Frequency**

**Intensity**

**Time**

**Type**
Week 1–23 day/weekSelf-selected15 minWalkingWeek 3–43 day/weekSelf-selected20 minWalkingWeek 5–63 day/weekSelf-selected25 minWalkingWeek 7–83 day/weekSelf-selected30 minWalkingWeek 9–104 day/weekSelf-selected30 minWalkingWeek 11–125 day/weekSelf-selected30 minWalking

Participants received a printed physical activity booklet in the first PAC meeting containing basic information about stretching, warm-up and cool down routines and a printed physical activity log to record all physical activity sessions performed over the 12-week period of intervention according to the progressive goals established every meeting. The progressive goals were related to the increase in volume of physical activity (frequency and time) over the intervention. Walking was established as the type of physical activity because this is the most common and accessible type of aerobic exercise performed in Brazil [34]. It should be noted that aerobic exercise is the primary type of exercise recommended for hypertensive patients [4,5,35]. The approach used regarding intensity was self-selected exercise intensity. Self-selected exercise is characterized by individuals choosing their own exercise intensity according to their preferences and adults typically self-select exercise intensities close to their ventilatory threshold [36], which is within the recommended range proposed for hypertensive patients (i.e., ≥40% of heart rate reserve or oxygen uptake reserve) [5]. Moreover, self-selected exercise intensity has psychological advantages compared to imposed exercise intensities, such as higher perceived autonomy, self-efficacy, affect, and lower perceived exertion [37]. From visit two to six, all participants were instructed to reproduce the walking exercise during the PAC meetings at a self-selected pace on a treadmill with the time established in the previous meeting in order to assess the intensity reached during this pace. HR (Polar Electro^®^, Oy, Kempele, Finland) and rating of perceived exertion (RPE; Borg scale 6–20) [38] were monitored during these sessions.

Participants were additionally asked about barriers related to physical activity and possible support sources for exercise. Participants received information about the benefits of aerobic exercise for hypertension treatment. In addition, the physical activity plan was reinforced in every meeting with the participants and some advice was provided to them about how to maintain their physical activity plan in their daily routine. The control group received standardized instructions about physical activity for health promotion according to the FITT principle during only one 15-min. face-to-face meeting after the baseline assessments. In addition, all participants were instructed to maintain their eating habits during the study period.

### 2.8. Statistical Analysis

Data normality was tested using the Shapiro–Wilk test. Results are expressed as mean ± standard deviation, absolute and relative frequency. An independent sample t-test was used to compare the baseline characteristics between groups. A repeated measure analysis of variance (ANOVA) (i.e., weeks 2, 4, 6, 8, 10 and 12) was used to compare the HR and RPE over the six face-to-face PAC meetings. The Bonferroni’s post hoc was used to identify pairwise difference. A generalized linear model with linear or gamma distribution with an identity link was used to compare the outcomes post-intervention adjusted by their respective baseline values between the groups. This approach was used because the baseline values were not considered for allocation of participants in the groups. The linear or gamma distribution model for each variable was defined by the lower value of Akaike Information Criterion, which represents quality of the fit to the model. The normality of the residuals was verified by normal Q-Q plot. The model results were expressed as estimated marginal means (EMM), parameter estimates (β), and 95% confidence interval (CI). A two-tailed *p <* 0.05 was considered statistically significant for all analyses. Analysis were performed using IBM SPSS Statistics for Win/v.25.0 (IBM Corp., Armonk, NY, USA). Cohen’s d_s_ was used to calculate the effect size (ES) [39]. The following criteria for the interpretation of the ES were adopted: <0.50, small; 0.50 to 0.79, medium; and ≥0.80, large [40]. 

## 3. Results

Participants had similar characteristics at the baseline assessment (Table 1).

**Figure 2:**
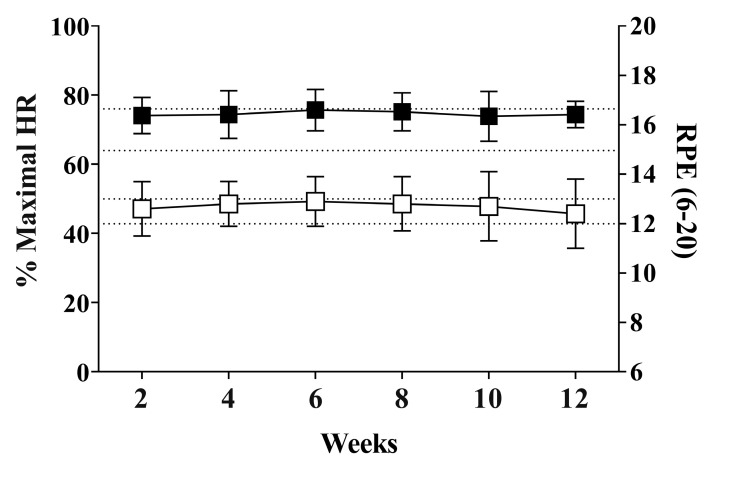
Heart rate (black square; left *Y* axis) and rating of perceived exertion (white square; right *Y* axis) responses during the 12-week physical activity counseling program. Dotted lines represent moderate intensity according to American College of Sports Medicine [33]: 64–76% of maximum heart rate (HR) and rating of perceived exertion (RPE) [12,13]. HR data of the participants under beta-blocker (*n* = 7) were not included due to its effect on HR. Data are expressed as mean ± standard deviation.

The HR (F(5, 15) = 0.132; *p* = 0.982) and RPE (F(5, 35) = 0.858; *p* = 0.519) responses during the self-selected walking exercise were not different over the six face-to-face PAC meetings (Figure 2). The HR responses varied from 74.1% to 75.7% of maximum HR and RPE responses varied from 12 to 13 on the Borg’s scale (6–20). The participants from the PAC group participated in all face-to-face counseling meetings. Only one adverse event was reported during the physical activity sessions, which was an ankle sprain, but the participant returned to the intervention one week later.

**Figure 3:**
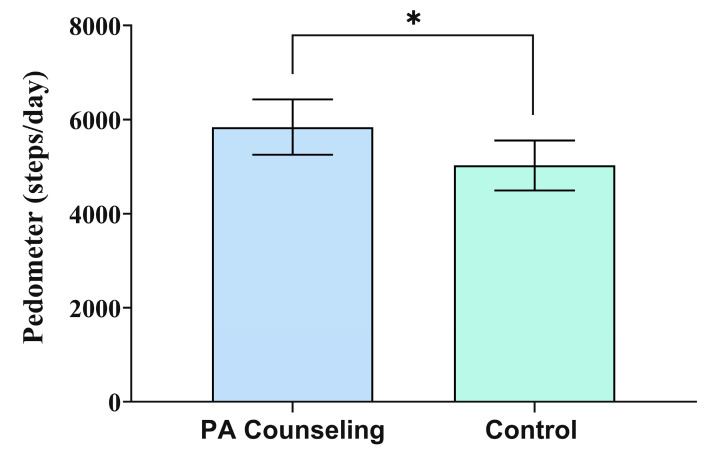
Pedometer-measured physical activity level of the participants from the physical activity (PA) counseling program group and control group at baseline and after 12 weeks. Data are expressed as estimated marginal means and 95% confidence interval. * Difference from the control group (*p* = 0.044) through the generalized linear model with linear distribution model adjusted by baseline value.

**Figure 4:**
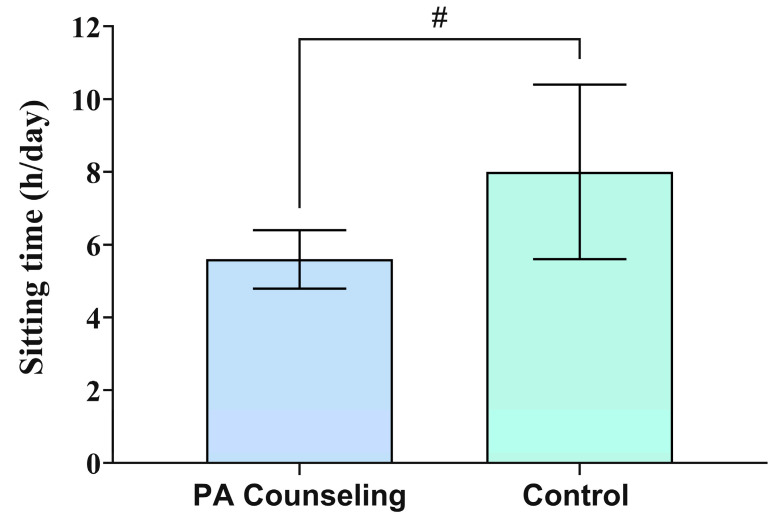
Sitting time of the participants from the physical activity (PA) counseling program group and control group at baseline and after 12 weeks. Data are expressed as estimated marginal means and 95% confidence interval. # Difference from the control group (*p* = 0.059) through the generalized linear model with gamma distribution model adjusted by baseline value.

Figure 3 shows the pedometer-measured physical activity level of the participants from the PAC and control groups at baseline and following 12 weeks. There was a difference between groups following 12 weeks of intervention (*W*(1) = 4.05; *p* = 0.044). The PAC group showed a higher number of steps per day compared to the control group (5839, 95% CI 5252, 6428 vs. 5028, 95% CI 4495, 5560, ES = 0.9; β = 811, 95% CI 21, 1600). Moreover, there was a trend for difference between groups following 12 weeks of intervention in the sitting time (*W*(1) = 3.55; *p* = 0.059) (Figure 4). The PAC group showed a trend for less time per day spent sitting compared to the control group (5.6, 95% CI 4.8, 6.4 vs. 8.0, 95% CI 5.6, 10.4, ES = 0.8; β = −2.4, 95% CI −4.9, 0.1) following 12 weeks of intervention.

Table 2 shows the resting and ambulatory BP levels of the participants from the PAC and control groups at baseline and following 12 weeks. There were no differences between groups following 12 weeks of intervention in resting (systolic, diastolic and mean; ps ≥ 0.133) and ambulatory (24-h, awake and asleep; ps ≥ 0.572) BP.

Table 3 shows the metabolic profile and body composition of the participants from the PAC and control groups at baseline and following 12 weeks. No differences between groups were found after 12 weeks for total cholesterol, HDL-cholesterol, LDL-cholesterol, and fasting glucose (ps ≥ 0.152). The control group showed lower triglyceride levels following 12 weeks compared to the PAC group (*p* = 0.003). The PAC group showed a lower waist circumference (*p* = 0.001) and a trend toward a lower quantity of abdominal fat (*p* = 0.059) compared to the control group following 12 weeks. No differences were observed for BMI, fat mass, and fat free mass between groups (ps ≥ 0.317).

## 4. Discussion 

The main findings of this pilot trial were: (i) 12 weeks of a PAC program based on the 5 A’s model modestly increased the pedometer-measured physical activity and showed a trend to decrease sitting time in hypertensive adults; (ii) this intervention did not change the BP status, metabolic profile, or body composition of the participants.

Our study showed that the PAC group increased ~800 steps per day compared to the control group. A previous systematic review [14] showed that medium- (from 31 min to 6 h of contact) and high-intensity (more than 6 h of contact, which is similar to the present study) PAC interventions are able to increase the physical activity level of healthy adults in ~40 min/week. However, the efficacy of counseling adults to increase physical activity level is still uncertain [41]. Kolt el al. [42] compared the effectiveness of 12 weeks of a standard time-based versus a pedometer step-based physical activity program involving one face-to-face meeting and three phone calls to the participants over three months in low active older adults. The participants performing the pedometer step-based physical activity intervention increased the leisure walking after three and 12 months; however, the participants from the standard-time based physical activity program also improved their physical activity level. Overall, pedometer-based physical activity interventions increase ~2000 steps per day [43,44]. A recent systematic review with meta-analysis conducted by Cavero-Redondo et al. [45] showed that an increase of 1000 steps per day in adults and older adults was associated with a 0.18 m/s decrease in pulse wave velocity, which is a marker of arterial stiffness, a subclinical risk factor for CVD [46]. Therefore, we do not rule out the possibility of some benefit to cardiovascular health such as a decrease in arterial stiffness elicited by a modest increase in pedometer-measured physical activity level, as observed in our study.

We included hypertensive adults with a sedentary lifestyle (<5000 steps per day) and low active lifestyle (5000 to 7499 steps per day) [29] in the present study. Although we observed an increase in the number of steps per day in the PAC group, these individuals did not change their physical activity status to an active lifestyle (7500 to 9999 steps per day) or to a highly active lifestyle (10,000+ steps per day) [29]. The goal in the last face-to-face PAC meeting for week 11 and 12 was to perform 30 min of self-selected walking exercise five times per week. Considering a moderate cadence (i.e., 60 steps per minute) [47], 30 min of walking is equivalent to 1800 steps. Therefore, it should be expected that the participants from the PAC group reached ~9000 steps per day in the last two weeks of intervention. However, we observed a mean value of ~6000 steps per day in the post-intervention assessment. This suggests that the participants from the PAC group did not maintain the established goal one week after the end of the PAC program, which could partially explain the absence of effects on the analyzed outcomes.

Regarding the BP status, we did not observe a reduction in resting and ambulatory BP (24-h, awake and asleep) following 12 weeks of intervention. A meta-analysis conducted by Bravata et al. [43] showed that a pedometer-measured increase in physical activity of ~2000 steps per day was associated with a 3.8 mmHg decrease in systolic BP. The magnitude of the decrease in BP was associated with greater baseline BP level and change in steps per day. Igarashi et al. [48] investigated the relationship between steps per day and changes in BP. The authors found a reduction of 3.1 and 1.6 mmHg in systolic and diastolic BP following pedometer-based physical activity interventions. An increase in 2000 steps per day was associated with a decrease of ~4 mmHg in systolic BP. A similar decrease in systolic BP was observed between interventions that achieved and did not achieve 10,000 steps per day (−3.1 vs. −3.3 mmHg). The intensity of physical activity is a key factor to determine BP reduction in a hypertensive population. Moderate intensity aerobic exercise training is able to elicit a significant BP reduction [5,6]. Interestingly, our data show that the participants exercised at a moderate intensity (i.e., 64–76% of HRmax, RPE 12–13) during the supervised treadmill trials over the six PAC meetings (Figure 2). Therefore, it seems that the absence of a reduction in BP status found in our study may also be associated with the modest increase in steps per day (i.e., ~800 steps per day) following the PAC program. In addition, we do not rule out the possibility that under no supervision, the participants exercised at a lower intensity than during the supervised treadmill trials in the PAC meetings.

As previously mentioned, the participants from the PAC program were instructed to reach 150 min/week of self-selected walking exercise in the last two weeks of the intervention. Therefore, it seems that a 12-week period is not enough to elicit a BP reduction when only a modest increase in steps per day (i.e., <1000 steps per day) occurred following a PAC program based on self-selected walking exercise in medicated hypertensive adults. It should be noted that considering both resting (<140/90 mmHg) and ambulatory (<130/80 mmHg) BP levels, the participants from our study were classified as controlled hypertensive adults. This aspect is important to be considered given that the magnitude of BP reductions following walking-based interventions is associated with baseline resting BP values [49,50]. Mandini et al. [49] found that participants with resting systolic BP above 160 mmHg reduced systolic BP by 21.3 mmHg following six months of a 300 min/week walking-based intervention, while participants with resting systolic BP between 120–129 mmHg reduced it by only 2.6 mmHg. Moreau et al. [50] found a strong correlation (r = 0.75) between the reduction in systolic BP following 24 weeks of a walking-based intervention and the baseline BP levels of the pre-hypertensive and hypertensive postmenopausal women. Taken together, it seems that a 12-week period of the PAC program, which results in a modest increase in steps per day, does not elicit a BP-lowering effect in medicated hypertensive adults with controlled BP levels.

Moreover, no significant improvement was observed in the metabolic profile and body composition of the participants from the PAC group compared to the control group following 12 weeks. Bravata et al. [43] showed that a pedometer-measured increase in physical activity of ~2000 steps per day was not associated with improvements in the metabolic profile of the individuals, although a modest decrease of 0.38 kg/m^2^ in BMI was observed. Similarly, Moreau et al. [50] did not find changes in the metabolic profile (i.e., fasting glucose and insulin) of pre-hypertensive and stage 1 hypertensive postmenopausal women following 24 weeks of a walking-based intervention. The authors found a modest decrease in BMI, but no changes in the percentage of body fat was observed. According to the American College of Sports Medicine position stand, 150–250 min/week of moderate physical activity is recommended to prevent weight gain, and more than 250 min/week is recommended to elicit a clinically significant weight loss in adults [51]. Therefore, the low volume of walking exercise associated with the instruction to maintain the dietary habits over the study may explain the absence of clinically significant changes in metabolic profile and body composition of the participants from the PAC group, although we observed a significant reduction in waist circumference and a trend to reduce abdominal fat in these participants. The control group showed lower triglyceride levels compared to the PAC group, which is unexpected. This finding can be due to other lifestyle changes not assessed in the present study, such as dietary habits.

Regarding the sitting time, which is a proxy of sedentary behavior, we found a reducing trend by ~2 h/day in the participants from the PAC group compared to the control group following 12 weeks. High sitting time has been associated with increased risk of CVD and all-cause mortality in adults, especially in less active individuals [52]. Stamatakis et al. [52] recently showed that each additional hour of daily sitting was associated with increased CVD for high sitters (i.e., >6 h/day), and all-cause mortality risk of 7% and 4%, respectively. However, replacing 1 h of sitting per 1 h of moderate physical activity was associated with a 20% lower CVD mortality risk in high sitters. In addition, replacing 1 h sitting for 1 h walking was associated with a 22% lower all-cause mortality risk in these individuals. Therefore, in order to improve the moderate to vigorous physical activity level as most guidelines recommend, it is also important to reduce the time spent in sedentary behavior, such as sitting time. The PAC group increased the steps per day and showed a trend to reduce sitting time, both important aspects related to a healthier movement behavior.

This study has limitations that should be mentioned. First, this study included medicated hypertensive adults aged 30–59 years without known CVD. Therefore, the observed results may be not directly translated to younger or older hypertensive populations with or without known CVD. Second, the participants of the present study showed controlled resting and ambulatory BP levels. Thus, we do not rule out the possibility that the PAC program is able to elicit a BP-lowering effect in hypertensive patients with uncontrolled BP levels. Third, although we instructed the participants to maintain their eating habits, no objective assessment of this aspect was conducted in the study. Fourth, the two groups were unbalanced in relation to sex (PAC: 18.2% males; Control: 36.4% males), which can make the interpretation of some outcomes, such as body composition, difficult. Despite the above-mentioned limitations, this pilot trial has reported results that should be considered in future randomized controlled trials designed to assess the efficacy and/or effectiveness of PAC programs to reduce BP levels in hypertensive adults.

## 5. Conclusions

Our preliminary findings show that 12 weeks of a PAC program based on the 5 A’s model increased the pedometer-measured physical activity level and showed a trend to reduce sitting time in hypertensive adults. However, the modest increase of ~800 steps per day and the trend to decrease ~2 h/day in sitting time were not associated with a reduction in BP and improvements in metabolic profile and body composition. Future randomized controlled trials should investigate the impact of longer PAC programs (i.e., >12 weeks) and/or counseling programs that aim to increase the physical activity level of the participants in a higher magnitude (e.g., >1000 steps per day) than that observed in our study.

## Figures and Tables

**Table 1:** Characteristics of the participants (*n* = 22).

	PA Counseling	Control	*p*
*N*	11	11	
Sex (♂/♀)	2/9	4/7	
Age (years)	49.6 ± 8.1	47.9 ± 6.7	0.592
Height (cm)	160.3 ± 8.3	162.0 ± 9.9	0.683
Body mass (kg)	85.5 ± 18.0	80.7 ± 12.7	0.483
Body mass index (kg/m²)	33.0 ± 5.3	31.3 ± 5.1	0.452
Waist circumference (cm)	97.0 ± 10.7	102.3 ± 12.6	0.298
Body fat (kg)	36.5 ± 9.0	32.5 ± 9.2	0.325
Body fat (%)	44.2 ± 4.0	41.0 ± 8.9	0.287
Abdominal fat (kg)	3.5 ± 1.2	3.0 ± 1.0	0.305
Fat free mass (kg)	47.2 ± 10.6	48.7 ± 9.7	0.728
Resting HR (bpm)	77.5 ± 8.0	74.7 ± 8.4	0.801
Resting SBP (mmHg)	130.5 ± 14.3	130.8 ± 13.9	0.964
Resting DBP (mmHg)	78.6 ± 8.8	85.4 ± 10.0	0.110
24-h SBP (mmHg)	123.9 ± 9.4	130.4 ± 14.1	0.221
24-h DBP (mmHg)	69.8 ± 6.6	79.9 ± 11.9	0.226
**Physical activity level**			
Pedometer (steps/day)	5237 ± 1526	5158 ± 1647	0.438
**Antihypertensive medication, *n* (%)**			
ACE inhibitor	1 (9.1%)	1 (9.1%)	
Diuretic	4 (36.3%)	4 (36.3%)	
Beta blocker	3 (27.2%)	2 (18.2%)	
Angiotensin receptor antagonist	7 (63.3%)	10 (90.9%)	
Calcium channel blocker	2 (18.2%)	0 (0%)	

Data are expressed as mean ± standard deviation. PA = physical activity; HR = heart rate; SBP = systolic blood pressure; DBP = diastolic blood pressure; ACE = angiotensin-converting-enzyme.

**Table 2:** Resting and ambulatory blood pressure of the participants from the physical activity (PA) counseling program group and control group at baseline and after 12 weeks.

	PA Counseling			Control					
	Baseline	Post 12-week	EMM (95% CI)	Baseline	Post 12-week	EMM (95% CI)	β (95% CI)	ES	*p* ^&^
**Resting BP**									
SBP (mmHg)	130.5 ± 14.3	123.3 ± 8.3	123.3 (119.1, 127.6)	130.8 ± 13.9	128.6 ± 13.5	128.6 (123.3, 133.9)	−5.3 (−12.0, 1.6)	0.65	0.133
DBP (mmHg)	78.6 ± 8.8	75.5 ± 9.1	77.6 (73.8, 81.3)	85.4 ± 10.0	83.1 ± 9.0	81.0 (76.1, 85.9)	−3.4 (−10.3, 3.4)	0.46	0.326
PP (mmHg)	51.9 ± 11.1	47.8 ± 6.8	46.1 (43.7, 48.7)	45.5 ± 8.8	45.5 ± 6.0	46.8 (44.4, 49.4)	−0.7 (−4.4, 2.9)	0.16	0.700
MBP (mmHg)	95.9 ± 9.6	91.4 ± 8.3	92.8 (88.8, 96.7)	100.5 ± 10.7	98.3 ± 10.3	96.9 (92.2, 101.7)	−4.1 (−10.4, 2.0)	0.56	0.865
**24-h BP**									
SBP (mmHg)	123.9 ± 9.4	120.4 ± 10.8	122.9 (118.1, 127.8)	130.4 ± 14.1	128.3 ± 14.3	124.9 (120.6, 129.2)	−2.0 (−8.8, 4.9)	0.26	0.572
DBP (mmHg)	69.8 ± 6.6	68.9 ± 7.5	73.0 (70.1, 76.1)	79.9 ± 11.9	79.0 ± 11.7	73.6 (70.5, 77.0)	−0.6 (−5.4, 4.1)	0.11	0.801
PP (mmHg)	54.1 ± 8.0	51.5 ± 7.2	49.8 (48.0, 51.4)	50.5 ± 4.7	49.3 ± 4.4	50.5 (48.7, 52.4)	−0.7 (−3.4, 1.9)	0.22	0.592
MBP (mmHg)	87.8 ± 6.7	86.1 ± 8.0	89.7 (86.2, 93.4)	96.7 ± 12.5	95.4 ± 12.4	90.7 (87.2, 94.4)	−1.0 (−6.5, 4.5)	0.16	0.723
**Awake BP**									
SBP (mmHg)	124.3 ± 8.6	121.2 ± 9.7	124.1 (119.1, 129.2)	132.7 ± 14.2	129.3 ± 13.4	126.1 (121.3, 131.1)	−2.0 (−9.6, 5.5)	0.24	0.598
DBP (mmHg)	70.3 ± 6.9	70.0 ± 7.8	74.5 (71.4, 77.8)	82.6 ± 12.3	81.1 ± 11.3	75.4 (72.2, 78.8)	−0.9 (−5.6, 3.7)	0.17	0.692
PP (mmHg)	54.0 ± 8.0	51.2 ± 7.3	49.6 (47.4, 52.0)	50.1 ± 5.1	48.5 ± 4.3	49.7 (47.5, 52.0)	−0.1 (−3.5, 3.2)	0.18	0.937
MBP (mmHg)	88.3 ± 6.4	87.1 ± 7.8	91.1 (87.4, 94.9)	99.3 ± 12.7	97.3 ± 11.9	92.4 (88.6, 96.3)	−1.3 (−7.0, 4.4)	0.20	0.658
**Asleep BP**									
SBP (mmHg)	121.8 ± 12.5	119.3 ± 12.1	119.2 (114.6, 123.9)	122.2 ± 12.7	121.0 ± 15.1	120.2 (115.5, 125.1)	−1.0 (−7.7, 5.7)	0.13	0.760
DBP (mmHg)	68.2 ± 7.3	66.1 ± 7.0	67.4 (64.3, 70.6)	71.3 ± 10.3	71.1 ± 12.0	68.8 (65.3, 72.5)	−1.4 (−6.4, 3.7)	0.25	0.592
PP (mmHg)	53.6 ± 8.2	53.2 ± 8.0	51.8 (49.3, 54.5)	50.9 ± 4.7	49.9 ± 5.0	50.8 (48.4, 53.4)	1.0 (−2.6, 4.6)	0.23	0.585
MBP (mmHg)	85.0 ± 10.5	83.8 ± 8.2	85.0 (81.0, 89.2)	88.2 ± 10.9	87.7 ± 12.9	85.8 (81.6, 90.1)	−0.8 (−6.8, 5.3)	0.11	0.812

Data are expressed as mean ± standard deviation, estimated marginal means (EMM), parameter estimates (β), and 95% confidence interval (95% CI). BP = blood pressure; SBP = systolic blood pressure; DBP = diastolic blood pressure; MBP = mean blood pressure; PP = pulse pressure. Cohen’s ds effect size (ES) (<0.50, small; 0.50 to 0.79, medium; and ≥0.80, large). ^&^ *p*-Value refers to generalized linear model with gamma distribution model adjusted by baseline value.

**Table 3:** Metabolic profile and body composition of the participants from the physical activity (PA) counseling program group and control group at baseline and after 12 weeks.

	PA Counseling			Control					
	Baseline	Post 12-week	EMM (95% CI)	Baseline	Post 12-week	EMM (95% CI)	β (95% CI)	ES	*p* ^&^
**Metabolic profile**									
Fasting glucose (mg/dL)	93.5 ± 16.7	91.5 ± 13.1	92.5 (85.8, 99.6)	97.3 ± 18.9	99.9 ± 22.0	97.4 (91.3, 103.8)	−4.9 (−13.9, 4.1)	0.44	0.285
Triglycerides (mg/dL)	187.4 ± 86.8	204.5 ± 95.1	197.3 (177.1, 219.9)	207.8 ± 97.8	170.6 ± 68.0	154.2 (135.6, 175.3)	43.1 (14.5, 71.8)	1.23	0.003
Total cholesterol (mg/dL)	204.7 ± 22.1	198.5 ± 48.3	198.1 (173.2, 226.5)	205.1 ± 31.8	197.0 ± 48.5	194.7 (176.1, 215.3)	3.4 (−29.5, 36.3)	0.09	0.841
HDL-cholesterol (mg/dL)	41.3 ± 7.5	51.8 ± 11.2	50.8 (44.8, 57.5)	39.2 ± 10.7	44.5 ± 15.8	44.0 (37.8, 51.3)	6.8 (−2.4, 15.8)	0.61	0.152
LDL-cholesterol (mg/dL)	121.7 ± 26.8	105.6 ± 48.1	106.1 (84.2, 133.6)	130.8 ± 38.4	118.4 ± 41.8	115.6 (97.5, 136.9)	−9.5 (−40.7, 21.7)	0.25	0.556
**Body composition**									
Body mass (kg)	85.5 ± 18.0	85.8 ± 17.8	81.7 (80.5, 82.9)	80.7 ± 12.7	81.6 ± 12.4	83.1 (81.9, 84.4)	−1.4 (−3.2, 0.4)	1.00	0.119
BMI (kg/m^2^)	33.0 ± 5.3	33.1 ± 5.2	31.9 (31.5, 32.4)	31.3 ± 5.1	31.2 ± 4.5	31.7 (30.7, 32.8)	0.2 (−1.0, 1.5)	0.13	0.739
WC (cm)	102.3 ± 12.6	101.0 ± 11.9	97.6 (96.3, 99.0)	97.0 ± 10.7	98.7 ± 12.4	100.8 (99.5, 102.0)	−3.2 (−4.9, −1.4)	1.49	0.001
Body fat (kg)	36.5 ± 9.0	36.9 ± 8.9	33.7 (32.7, 34.7)	32.5 ± 9.2	33.3 ± 8.8	34.0 (33.5, 34.8)	−0.3 (−1.7, 0.8)	0.72	0.526
Body fat (%)	44.2 ± 4.0	44.7 ± 4.2	42.9 (42.3, 43.5)	41.0 ± 8.9	41.6 ± 8.3	42.4 (41.6, 43.2)	0.5 (−0.5, 1.5)	0.43	0.317
Abdominal fat (kg)	3.5 ± 1.2	3.5 ± 1.1	3.1 (2.8, 3.3)	3.0 ± 1.0	3.3 ± 1.2	3.4 (3.2, 3.5)	−0.3 (0.1, −0.6)	1.00	0.059
Fat free mass (kg)	47.2 ± 10.6	46.8 ± 10.8	46.6 (45.9, 47.3)	48.7 ± 9.7	48.7 ± 10.3	47.0 (46.5, 47.5)	−0.4 (−1.3, 0.4)	0.35	0.327

Data are expressed as mean ± standard deviation, estimated marginal means (EMM), parameter estimates (β), and 95% confidence interval (95% CI). BMI = body mass index; WC = waist circumference. Cohen’s ds effect size (<0.50, small; 0.50 to 0.79, medium; and ≥0.80, large). ^&^ *P*-value refers to generalized linear model with gamma distribution model adjusted by baseline values. Bold values indicate significance at *p* < 0.05.
